# Behavioural synchrony between fallow deer *Dama dama* is related to spatial proximity

**DOI:** 10.1186/s12862-021-01814-9

**Published:** 2021-05-06

**Authors:** Zoe E. Hoyle, Rosie A. Miller, Sean A. Rands

**Affiliations:** School of Biological Sciences, University of Bristol, Bristol, BS8 1TQ UK

**Keywords:** Behavioural synchrony, Group living, Copying, Active behaviour

## Abstract

**Background:**

Animals living in social groups can benefit from conducting the same behaviour as other group members. If this synchronisation is achieved by copying the behaviour of other individuals, we would expect synchrony to be more likely when pairs of individuals are close together.

**Results:**

By comparing the behaviour of a focal individual with its nearest, second nearest and third nearest neighbour and a control individual, we show that pairings of fallow deer *Dama dama* are more likely to be active or inactive at the same moment in time if they are closer together. We also demonstrate that synchronisation in the group happens more often than would be expected by chance.

**Conclusions:**

Our findings suggest that there is a relationship between the synchronisation of behaviour and the spatial proximity of individuals. Spatial proximity is likely to be an important influence on how likely individuals are to be synchronised, although care needs to be taken to separate social and environmental influences on individual behaviour.

**Supplementary Information:**

The online version contains supplementary material available at 10.1186/s12862-021-01814-9.

## Introduction

Many animal species spend all or part of their lives in social groups with other members of their species [1, 2]. Living together brings benefits to individuals, but it is likely that the behaviour that most individuals show within the group involves elements of conformity or response to the behaviour of other group members. In order to remain together as a group, it is likely that group members will have to conduct similar activities (such as moving or foraging) at the same time [3], and much theoretical work has been conducted that considers how collective decision-making can emerge from individuals within the group altering their behaviour in response to the behaviours of other group members [4–9]*.*

Behavioural synchrony is one process that can drive group decision-making [9, 10]. Behavioural synchronisation occurs when animals in a group perform the same behaviour as each other at the same moment in time (so focussing on what [11] describe as activity synchrony), and the degree of synchrony seen will be reflected by the proportion of time that individuals conform in their behaviour (see [12] for more discussion of how we can measure this, and what it is that we are actually measuring). Behavioural synchrony is often seen in sexually segregated species, and is suggested to be a result of sexually dimorphic males and females having differing energetic requirements and activity patterns (otherwise known as the activity budget hypothesis, discussed in detail in [9, 13–15]). However, groups (or species) don’t have to show sexual segregation to synchronise their behaviour, and synchronous behaviour may be important for ensuring that individuals remain well-fed and safe [16–18], as well as offering a coordinated vigilance [19, 20] or departure [21–24] strategy to the group.

Synchrony could be coordinated by one or several leaders or other decision-makers [25, 26], but these influential individuals are not necessary for synchronisation to occur [16], and it may be difficult for a few individuals to coordinate the actions of groups containing many individuals. In the absence of a leader or other decision-maker, synchrony could be achieved by paying attention to nearest neighbours [5], suggesting that spatial proximity may be influential in causing and maintaining synchrony. For example, some species of gull show temporal synchronisation of vigilance and sleep/resting behaviour when flocking [27–30]. Although this behaviour could be coordinated by one or several individuals 'leading' the flock's behaviour, the size of the flock and the spatial distribution of individuals within the flock means that it is more feasible to assume that individual birds are only able to observe and respond to their immediate neighbours. Given that many studies of large-scale group behaviour suggest that the behaviour of the group is driven by the interactions of local neighbours [31–33], this suggests that any synchrony of behaviour seen should be related to distance between individuals [30], and behavioural choices such as time spent searching for food or being vigilant for social interactions or predators may depend upon how close a neighbour is [34–36].

We would therefore expect individuals who are close together to be more synchronised than those that are separated by a greater distance. Previous observations of cows *Bos taurus* [37], red deer *Cervus elaphus* [10], and black-headed gulls *Chroicocephalus ridibundus* [30] have shown that this relationship can occur: pairs of individuals were more likely to be behaving similarly, the closer they were to each other (so a focal individual was more likely to be conducting the same behaviour as its immediate neighbour, and less likely to be conducting the same behaviour as its second closest neighbour, etc.). Similarly, a study of ponies *Equus caballus* showed that the individuals in a stable herd that tended to be closer to each other were also more likely to be synchronised [38]. Here, we consider how spatial proximity influences synchronisation in fallow deer, *Dama dama*, a highly social ungulate [39–41]. Using a similar technique to that used for red deer [10], we suggest that there is a relationship between synchronisation of behaviour and spatial proximity.

## Results

**Fig. 1:**
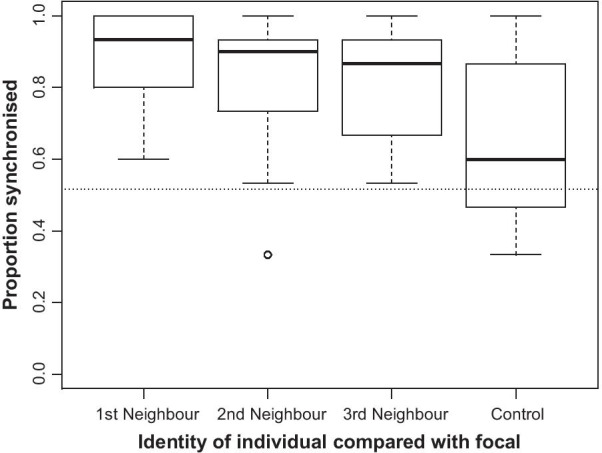
Proportion of samples where the focal individual is either active or inactive at the same time as the closest, second closest, third closest or a randomly sampled individual. Dotted line shows the expected level of synchronisation (0.516—see text) if all individuals are behaving independently of each other. All measures of synchronisation differed from each other (all pairwise post hoc comparisons *p* < 0.001)

Activity synchronisation between pairs of deer differed according to the amount of social separation between the focal and the individual with which it is being compared; this was significant both when behaviours are strictly classified according to the ethogram ($\chi_{3}^{2}$ = 27.8, *p* < 0.001, all post hoc pairwise comparisons *p* < 0.009) or when dichotomously classified as either active or inactive ($\chi_{3}^{2}$ = 41.8, *p* < 0.001, Fig. 1, all post hoc pairwise comparisons *p* < 0.001). The focal individual was more synchronised with its closest neighbours than with a random (control) individual in the herd, and this synchrony was greatest with the closest, and then the second closest, and then the third closest neighbour (Fig. 1). Potential pseudoreplication caused by multiple measurements from the same individual was unlikely to have influenced our results, given that none of the 100,000 tests conducted with resampling approached non-significance (where *p* < 0.0011 for all tests with the dichotomous classification of behaviour, and *p* < 0.0156 when strictly classified according to Table 1).

**Table 1:** Ethogram of individual behaviours recorded from instantaneous scan samples of fallow deer

Behaviour	Description	Coding
Drinking	Consuming water or other liquids	Active
Grazing	Standing or walking whilst eating, with head to the ground	Active
Interacting	Physical contact between two or more individuals, including intercourse, fighting or grooming	Active
Licking	Licking the surface of the body	Active
Nursing	Feeding a calf from their teat	Active
Resting	Laying down with head down	Inactive
Resting (vigilant)	Laying down with head raised off the ground	Inactive
Resting (chewing)	Laying down with head up, chewing	Inactive
Running	Moving at a fast pace	Active
Scratching	Itching itself using either a foot or its head	Active
Standing (vigilant)	Standing stationary with head up, looking alert	Active
Standing (chewing)	Standing stationary with head up, chewing	Active
Suckling	Sucking on the teat of an adult	Active
Walking	Moving at a regular slow pace	Active

If we assumed that the actions of all the individuals observed were independent of both previous behaviour and the behaviour of other individuals, then we would expect an individual deer to be active for proportion 0.411 of the time (as 924 of the 2250 individual observations made were active behaviour). Given this assumption of independence, we would expect any two individuals to be observed as either active together or non-active together in 0.516 of observations (given that if an individual is independently active for a proportion *a* of the total time and inactive for 1 – *a*, we would expect pairs of independently behaving individuals to be either active or inactive at the same time for *a*^2^ × (1 – *a*)^2^ of the total time). All median values given in Fig. 1 are greater than this value, which would suggest that neighbouring individuals are paying attention to the behaviour of others and that synchrony is not simply occurring due to chance alone. However, the simultaneous behaviour of the focal and control individuals were not significantly different from the predicted proportion of time conducting the same behaviour despite behaving independently ($\chi_{1}^{2}$ = 3.33, *p* = 0.068).

## Discussion

Our study demonstrates that spatial proximity is likely to be important for behavioural synchronisation in fallow deer, with individuals tending to synchronise their behaviour with closest individuals, echoing results seen in cows [37], black-headed gulls [30], and red deer [10]. Behavioural synchrony between individuals was common across all measured fallow deer pairs (Fig. 1). In another study of fallow deer recording the proportion of time mixed- or single-sex groups were completely synchronous or not, Villerette et al. [42] recorded that all-female groups were highly synchronous, and conformed totally within the group for 93.1% of the time. This value falls in the same region as the synchrony seen in the current study between the focal and its close neighbours, but is a little high when considering the action of a randomly selected control, which is likely to be due to differences in measurement criteria and definitions of group membership.

Because breeding males were not present in the observed groups, the individuals recorded here were likely to be similar in their activity budgets, leading to synchrony according to the activity budget hypothesis [9, 13–15]. Nonetheless, individuals may still have differed in their energetic requirements and hence synchrony due to being different sizes (e.g. [43]) or in different reproductive states [44, 45]. Similarly, dominance hierarchies could have influenced how likely spatially-close individuals were to synchronise [6, 17], and there could have been interactions between size, age, condition and dominance (e.g. [46, 47]*.*). Density of neighbours may also influence synchrony of behaviour ([48, 49], but see [50]). The study presented here did not measure individual size, spatial separation, or energetic state and was unable to identify long-term relationships between the unmarked animals, but it may be fruitful in future studies to do so.

Observing that close neighbours are more synchronised than distant individuals does not imply that close individuals are copying each other’s behaviour. It is plausible that observed synchronisation of behaviour within the group could be a result of all individuals responding to an immediate environmental cue in the same way, or instead be a result of multiple individuals behaving according to internal *zeitgebers* rather than their immediate social environment [51, 52]. The data we collected were not suitable for testing whether non-social stimuli were influential in driving synchronous behaviour, and it is difficult to disentangle whether a social or non-social cue drives synchrony, as they lead to the same hypothesised behaviour between closely-associated members of the group. If a specific environmental cue was hypothesised to generate the behaviour (such as heterogeneity in forage availability in the environment that causes individuals associated in the same area to spend more time focussed on feeding), this could potentially be manipulated to test whether spatial synchrony is altered. Alternatively, carefully-targeted manipulation of individuals (by supplementing or withholding food) would alter their foraging requirements independently of other group members, which would potentially lead to predictable directions of behavioural change in their immediate neighbours. This would manipulate subsets of the herd rather than the entire herd itself, and would potentially provide some evidence of copying behaviour being the mechanism causing synchronisation.

An individual’s spatial position within the group could influence the behaviour that it conducts, as its position may have effects upon its exposure to predators, its immediate ability to forage, or the degree of social interactions it has to engage in [53–55]. This means that individuals who are spatially close within the group will be experiencing similar environmental and social pressures, and may therefore be more likely to conduct similar behaviours in response to these pressures, showing more similarity among themselves than to an individual selected randomly from elsewhere in the group. The observational techniques used in the current study are not sufficient to disentangle environmentally-driven similarities in independent behaviour from copying, and experimental manipulations would be necessary to identify the mechanism causing synchrony [30]. Similarly, if synchronisation occurs because particular individuals within the group have influence over the behaviours of others (such as through dominance, leadership or some other aspect of the individuals, as described above), a closer study of how conformity spreads within the group would be necessary to disentangle the mechanism driving synchrony. This is particularly true if an individual's response represents a non-additive relationship between the metric distance and the degree of influence of a neighbour, which could in turn mean that distance between all the members of a group is not sufficient to explain how conformity behaviour spreads within the group. Careful analysis of video footage (ideally taken from above rather than the side, such as in [56]) would allow us to visualise the process, while careful manipulation of the social stimuli individuals receive (e.g. [57]*.*) would help to disentangle the interactions between distance and neighbour identity.

## Conclusion

This paper demonstrates that behavioural synchronisation may be tied to social proximity in fallow deer, echoing a similar result seen in closely related red deer. Although the mechanism driving this synchrony could not be identified using the technique presented here, our results suggest that there is a relationship between the spatial proximity of individuals and the synchronisation of their behaviour. Further studies on other aggregating species are urged: the technique presented here is simple to implement, and would be suitable for demonstrating spatially-organised synchronisation in a wide range of socially-aggregating species.

## Methods

The study was conducted in Ashton Court Estate, Bristol, UK (51º26′34″ − 2º38′52″) during Autumn 2018. This is a public space, and no permission was required to make behavioural observations. The deer live in a managed herd of about seventy individuals, within a fenced park consisting of managed mixed woodland, open grassland and bracken. Rands et al*.* [10] describe the location in detail; note that the fallow deer are fenced in a separate area to the managed herd of red deer described in that paper*.* A pilot study was initially conducted to identify the behavioural repertoire of deer within the herd (Table 1), and to ensure that the observers were consistent in identifying these behaviours. All observations were conducted by RAM and ZEH from ad hoc locations outside the fenced deer park, and the observers did not move from their positions during the sampling period (and were in location at least five minutes before each sampling period, to allow for habituation). Access to the deer park was restricted during the study period, but deer were habituated to the continuous presence of casual walkers, bicycles, small motor vehicles and dogs immediately outside their fenced area. Observations were conducted when the deer were collected (and visible to the observers) in an open grassland area of the enclosure (interspersed with a few mature trees with no branches at deer height), where it was assumed the deer would have unimpeded visual contact with each other and where all deer could be seen by the observers. Because of the density of the population within the restricted space of the deer enclosure, we assumed that the herd members were collected in a single dispersed group.

Data collection followed the protocol detailed in [10]. A focal individual was randomly selected from the deer visible to the observers using a random number generator. Male fallow deer (bucks) possessing antlers were excluded from selection and were not recorded in the observations, as they were likely to be spatially distant from the main herd due to sexual segregation behaviour resulting from rutting behaviour [58] (and males and females are typically spatially segregated during daylight [40, 41], and show differing vigilance behaviours [59]). The instantaneous behaviour (as described in Table 1) of this focal individual was then recorded every 60 s after the selection moment, for 15 min. At the same time, the behaviours of the nearest, second nearest, and third nearest individual to the focal were recorded (noting that the identity of these neighbours could change as individuals moved closer to or further away from the focal individual). The spontaneous distance used to allocate identities to deer was estimated using observable body lengths, which were visually compared by observers during an observation. At the same time, a control individual was randomly selected from all the visible individuals (excluding the focal and its three closest neighbours), and its behaviour was also recorded. The identity of the control individual was randomised at every observation, meaning that the control represented the mean behaviour performed by all of the visible individuals. During a series of observations, one observer stayed focussed on the focal individual and its neighbours, whilst the other observer scored the control behaviour and wrote down all of the data. These roles were swapped between RAM and ZEH between sets of observations.

Thirty sets of fifteen minute observations were collected on five days between 17 and 29th October 2018, starting no earlier than 11.58 (GMT) and ending no later than 16.48 during daylight hours. Unlike the comparable datasets collected in [10] and [30], there was no need to abort data collection at any point due to disturbance. Collecting data over this short window of time ensured that deer were behaving similarly, and had not changed their social behaviour in response to changes in reproductive status or changing temperature, light levels or environmental conditions caused by seasonal change.

Using the strict descriptions of instantaneous observed behaviour described in Table 1 may falsely classify animals that are conducting synchronised behaviours that are masked by transient additional behaviours (so, in our classification an animal that is resting but vigilant is classified as conducting a different behaviour to one that is resting and chewing). To avoid these fine-scale mistakes, we also categorised the data into a dichotomous descriptor ‘inactive’ (when the deer were lying on the ground) or ‘active’ (all other activities; Table 1), following a system previously used for both fallow deer [42] and red deer [10]. We calculated the number of times the first-, second- and third-closest neighbour and the control individual were active or inactive at the same time as the focal individual for each fifteen-minute observation sample (note that proportions are used for plotting the figure). Similarly, we calculated the number of times these pairings were conducting the same strict behaviour as defined in Table 1. None of these data were normally distributed, and so we conducted a Friedman test to see whether there were differences in synchronisation between the four types of individual for both strict and dichotomised comparisons, using *R* 3.6.1 [60]. Post hoc pairwise comparisons were conducted using the test suggested by Conover [61] with Bonferroni corrections, implemented using *PMCMR* 4.3 [62]. To test whether the synchronisation between control and focal individual differed from the predicted value when behaving independently, we conducted a one-sample signed-rank test, considering the predicted ‘independent synchrony’ proportion (0.516) as the hypothesised median. The full dataset and *R* code used for all analyses are available in Additional file 1, Additional file 2, Additional file 3.

As noted by [10], pseudoreplication is likely and unavoidable when measuring group synchrony behaviour by sampling unmarked individuals. This could be counteracted by making the sample size small enough for pseudoreplication to be unlikely, but given the herd size in this study, this was not an option available here. The unit of replication is the focal individual rather than its neighbours or the control, but, given that we randomly chose our focal individual thirty times from a population of about seventy individuals, it is likely that at least a few of these focal individuals were actually the same deer, potentially biasing the statistical analyses of our results in an unpredictable direction. To explore whether any pseudoreplication could skew our dataset, we generated 100,000 datasets where each of the thirty sample sets collected in our observations had a focal deer identity randomly allocated to it, assuming that there were seventy deer in the herd. The random allocation assumed that all seventy individuals were equally likely to be chosen, and that each sample was independent of the others in the dataset, meaning that a dataset could have several samples which were allocated the same focal deer. Having allocated focal identities, the dataset was then filtered so that each focal individual was only sampled once, which was done by identifying which focal individuals had multiple samples allocated to them and randomly removing all but one of the sample. A Friedman test was conducted on each of the 100,000 resampled filtered datasets, and the significance values were recorded for all of these.

## Supplementary Information

**Additional file 1.** Text file containing raw data, with a description of the coding given in the header of the file.

**Additional file 2.** Text file containing processed dataset, for use in *R* code.

**Additional file 3.** *R* code for analyses.

## Data Availability

The dataset supporting the conclusions of this article is included within the article and its additional files.
